# Clinical outcomes observation in stage IIB–IIIB cervical cancer treated by adjuvant surgery following concurrent chemoradiotherapy

**DOI:** 10.1186/s12885-021-08146-3

**Published:** 2021-04-21

**Authors:** Yong Li, Zhiying Chen, Xiang Wang, Xiumei Li, Jie Zhou, Yongchun Zhang

**Affiliations:** 1 Department of Gynecology, The Affiliated Hospital of Qingdao University, Shandong 266003 Qingdao, China; 2 Department of Radiation Oncology, The Affiliated Hospital of Qingdao University, 16 Jiangsu Road, Qingdao, 266003 P. R. China; 3 Department of Abdominal Ultrasound, The Affiliated Hospital of Qingdao University, Shandong 266003 Qingdao, China

**Keywords:** Cervical cancer, Concurrent chemoradiation, Surgery, Recurrence

## Abstract

**Background:**

To explore the feasibility of adjuvant surgery following concurrent chemoradiation therapy (CCRT) in stage IIB–IIIB (according to FIGO staging of 2009) cervical cancer and analyze risk factors of recurrence after surgery.

**Methods:**

Forty-nine patients diagnosed with stage IIB–IIIB cervical cancer were reviewed retrospectively. We investigated the risk factors of recurrence after surgery using Chi-squared Test and further analyzed multiple factors affecting postoperative recurrence using the multi-factor logistic regression. Furthermore, the correlation of surgery outcomes (including operation time, bleeding, and hospitalization date and surgery complications) with the time which carried out between CCRT and completion surgery was analyzed.

**Results:**

Tumor histology and residual tumor in the cervix were significantly associated with postoperative recurrence (*P* = 0.014 and *P* = 0.040, respectively). Logistic regression analysis demonstrated that the independent risk factors of postoperative recurrence were age and residual tumor in the cervix (*P* = 0.017 and *P* = 0.030, respectively). Complications (operation time, bleeding, hospitalization date) were compared between patients with an interval with radiotherapy less than 6 weeks and patients with an interval longer than 6 weeks. There were statistical differences in the amount of bleeding and postoperative complications between the two groups (*P* = 0.019 and *P* = 0.044, respectively).

**Conclusion:**

CCRT combined with surgery for stage IIB–IIIB cervical cancer was feasible, reduced the rate of postoperative recurrence and surgery complications were tolerated.

## Background

Cervical cancer is a worldwide major public health issue. It is the second most common malignancy in women and represents the third-leading cancer in women worldwide [1]. Approximately 500,000 new cases and 237,500 deaths of cervical cancer occur annually. Advanced cervical cancer is not easy to control and has poor prognosis because of the lymph node metastasis or distant metastasis. Cisplatin- based concurrent chemoradiotherapy is the standard treatment for advanced cervical cancer, but the local recurrence rate after chemoradiotherapy is high and 5-year survival rate is only 50 to 65% [2]. In order to further improve the local control rate of cervical cancer and improve the 5-year survival rate, many scholars have explored the efficacy and safety of concurrent radiotherapy and chemotherapy combined with surgery in the treatment of advanced cervical cancer [3]. Some studies have reported that CCRT combined with surgery can improve local lesion control and overall survival, but the role of adjuvant surgery is still controversial, because these studies differ in disease stage, surgical scope, and form of CCRT (radiotherapy dose, internal irradiation or not, and type of chemotherapy) [4]. The purpose of this paper is to explore the feasibility of CCRT joint surgery in cervical cancer patients with IIB–IIIB period and retrospectively analyze the related factors of postoperative recurrence.

## Methods

### Patients

This study included 49 patients with biopsy proven cervical cancer between June 2006 and October 2012 was treated in department of Radiotherapy Center and Gynecology of the Affiliated Hospital of Qingdao University. The Institutional Review Board approved this study and informed consent was obtained. Inclusion criteria: a) Eastern Cooperative Oncology Group (ECOG) status was less than or equal to 1; b) International Federation of Gynecology and Obstetrics (FIGO) stages IIB–IIIB cervical cancer through gynecological examination by two veteran gynecologic oncologists; c) All patients underwent a medical history, gynecological examination; punch biopsy, chest X-ray, pelvic magnetic resonance imaging (MRI) and transvaginal ultrasound (TVS). Exclusion criteria: a) Patients with serious heart, lung, liver or hematologic system disease; b) Patients with other cancer; c) Treatment was interrupted or radiotherapy did not reach the radical dose; d) Lost to follow-up or death from other causes.

### Concurrent chemoradiation therapy (CCRT)

All patients completed the radical radiotherapy in department of Radiotherapy Center. Patients were immobilized with a body net in the supine position and underwent a computed tomography (CT) simulation scan (General Electric, Milwaukee WI, USA) with intravenous contrast, using 5 mm slice thickness. Simulation images extended from L1 to 5 cm below the ischial tuberosities. The gross tumor volume (GTV), clinical target volume (CTV) and planning target volume (PTV) were defined according to Radiation Therapy Oncology Group (RTOG) guidelines. The CTV-high risk included the GTV, parametrium, the upper part of the vagina to 3 cm below the tumor invasion, and metastasis lymph node. The CTV-low risk included the CTV-high risk and regional lymph nodes (common, external, internal iliac lymph nodes, obturator and presacral lymph nodes). The treatment planning was designed and computed using the Plato system version 2.7.5 (Varian, USA). The external radiation dose was 40–50 Gy in 20–25 fractions with 2 Gy per fraction using a linear accelerator (Trilogy, Varian, USA). And followed by high-dose-rate brachytherapy to 90% of the high-risk CTV was delivered with 30–40 Gy using an intracavity applicator (microSelectron-HDR Ir-192 set; Nucletron, Veenendaal, Netherlands). All patients received concomitant chemotherapy of cis-platinum (40 mg/m^2^) alone every week starting with external radiotherapy (4–5 circles).

### Adjuvant surgery

After reevaluated the relationship between cervix, bladder, rectum and parauterine tissue through gynecological examination and pelvic MRI, all patients underwent extrafascial hysterectomy with adnexectomy followed by CCRT. According to postoperative pathology, nine patients with invading ½ of the cervical stroma recived 4 circles chemotherapy consisted of the association of cis-platinum (100 mg/ m^2^) in combination with paclitaxel (135 mg/ m^2^).

### Follow-up

Patient follow-up was designed to be conducted every 3 months during the first 2 years and every 6 months over the next 3 years after surgery. The follow-up included gynecological examination, TVS, vaginal apical cytology, abdominal plevic and chest CT.

### Statistical analysis

All data were performed using SPSS 20.0 (Chicago IL, USA). Quantitative data are expressed as the mean ± standard deviation (S.D.). χ^2^ test or *Fisher* exact test was used to evaluate categorical variable. Groups were compared using Student’s *t*-test.. Multivariate analysis of prognostic factors was performed with Cox proportional hazards regression. A *p* < 0.05 was regarded as statistically significant.

## Results

The correlations of major clinical/pathological factors and recurrence after CCRT and adjuvant surgery in LACC were summarized in Table 1. Median age of 49 patients was 49 years (range 29–66) and there was statistically significant difference in age and recurrence of disease between two groups (*P* = 0.023). Median follow-up from the date of surgery was 76 months (range 29–129 months), and 5 patients died during follow-up. The 3-year OS and 5-year OS were 95.9 and 89.8%, respectively.

**Table 1:** Correlations of major clinical/pathological factors and recurrence after CCRT and adjacent surgery in stage IIB–III cervical cancer

Factors		Number (%)	Recurrence		χ^2^	*p*
			No	Yes		
Age	<50	28(57.1)	50	3	5. 168	0.023*
	≥50	21(42.9)	13	8		
HPV infection	No	31(63.3)	25	6	0.464	0.496
	Yes	18(36.7)	13	5		
Tumor diameters	≤4 cm	20(40.8)	14	6	1.107	0.293
	>4 cm	29(59.2)	24	5		
Pathological differentiation	High	7(14.3)	5	2	0.979	0.613
	Moderatly	33(67.3)	25	8		
	Low	9(18.4)	8	1		
Pathologic subtypes	Adenocarcinoma/mixed subtypes	7(14.3)	4	3	1.954	0.162
	SCC	42(85.7)	34	8		
FIGO stage	IIB	41(83.7)	32	9	0.657	0.720
	IIIA	1(2.0)	1	0		
	IIIB	7(14.3)	5	2		
Microscopic residual	No	27(55.1)	20	7	8.920	0.012*
	Disease invading less than 50% of the myometrium	13(26.5)	13	0	8.920	0.012*
	Disease invading more than 50% of the myometrium	9(18.4)	5	4		

*CCRT* concurrent chemoradiotherapy, *HPV* human papillomavirus, *FIGO* International Federation of Gynecology and Obstetrics, *SCC* squamous cell carcinoma, *P<0.05

In early postoperative period (≤ 30 days), 2 incomplete intestinal obstruction and 3 deep venous thrombosis were found. As far as long-term postoperative period (> 30 days), 3 complications were observed: 2 patients developed colorectal fistula and 1 patient showed pelvic pain. The average of operation time was 2.0 ± 0.4 h (range 1.2–3.1 h) and blood loss during the surgery was 133.5 ± 66.7 mL (range 50–400 mL). The average hospital stay was 9.4 ± 2.1 days (range 6–15 days). The relationship between the time to start operation after CCRT and blood loss during the surgery, hospital stay and postoperative complication were analyzed furthermore (Table 2). Although blood loss during the surgery, hospital stay and postoperative complication in > 6 weeks group were less than that in ≤6 weeks group, only blood loss during surgery and postoperative complication were closely related with the time to start operation after CCRT (F = 5.866, *P* = 0.019; F = 4.056, *P* = 0.044).

**Table 2:** The evaluation of operation time

Groups	Operation time (h)	*p*	Bleeding (mL)	*p*	Hospital stay (day)	*p*	Complications		*p*
							No	Yes	
≤6 weeks	1.812 ± 0.376	0.942	147.41 ± 78.377	0.019*	9.740 ± 2.141	0.225	20	7	0.044*
>6 weeks	1.944 ± 0.368		115.70 ± 43.425		9.050 ± 2.081		21	1	

*P<0.05

After the combination of CCRT and surgery, recurrence of disease was observed in 12 (22.4%) patients, which were distributed as follows: 9 local recurrences and 3 distant metastases (1 lung metastasis and 2 bone metastases). After adjuvant surgery, 27 patients (55.1%) showed a complete response to CCRT treatment, and 22 cases (44.9%) showed a microscopic residual disease (9 cases with disease invading more than 50% of the myometrium and 13 cases with disease invading less than 50% of the myometrium). There was statistically significant difference in recurrence of disease and microscopic residual (*P* = 0.012). On multivariate analysis showed both age and microscopic residual were highly correlated with recurrence of disease (Table 3) (*P* = 0.017 and *P* = 0.030, respectively). Although there was not statistically significant difference in tumor diameters and recurrence of disease, tumor diameters was highly correlated with microscopic residual on further analysis (Table 4).

**Table 3:** Multivariate analysis of recurrence after CCRT and adjacent surgery in stage IIB–III cervical cancer

Parameters	*P*	Exp (*B*) 95% *CI*
Age	0.017*	25.086(1.777 ~ 354.179)
HPV infection	0.271	3.633(0.365 ~ 36.167)
Tumor diameters	0.765	0.715(0.079 ~ 6.476)
Pathological differentiation	0.487	3.168(0.123 ~ 81.769)
Pathologic subtype	0.339	0.205(0.008 ~ 5.296)
FIGO stage	0.252	0.080(0.001 ~ 6.008)
Residual cancer	0.030*	0.038(0.002 ~ 0.725)
Constant	0.544	

*CCRT* concurrent chemoradiotherapy, *P<0.05

**Table 4:** The relationship between macroscopic residual and clinical/pathological factors

clinical/pathological factors		Macroscopic residual		χ^2^	*p*
		No	Yes		
Tumor diameters	≤4 cm	18	2	14.337	<0.001*
	>4 cm	9	20		
Pathological differentiation	High	4	3	0.017	0.992
	Moderately	18	5		
	Low	5	4		
Pathologic subtype	Adenocarcinoma/mixed subtypes	3	4	0.086	0.769
	SCC	24	18		

*SCC* squamous cell carcinoma, *P<0.05

## Disscussion

Local and distant recurrence is the main reason for the failure of treatment of locally advanced cervical cancer. Studies have reported that CCRT can improve the local control of lesions, progression-free survival and total survival in patients with locally advanced cervical cancer. Some researchers [5, 6] believe that the control of local lesions can improve the surgical resection rate. At present, there are few studies on adjuvant surgery, which is controversial for further reducing recurrence rate and improving prognosis. In this study, a total of 49 patients with locally advanced cervical cancer were included. The lesions all shrank to different degrees after CCRT treatment, among which 18 patients showed significant improvement in paratactic infiltration. About 3–9 weeks after CCRT treatment, all patients underwent extrafascial hysterectomy and bilateral adnexectomy, and the incidence of postoperative complications was 16.3%, similar to 19.8% in the previous study [7]. 9 cases of patients were proved to be local recurrence with pathologically, and distant metastasis was observed in 3 patients. The recurrence rate (22.4%) was little higher compared with the previous work, which 192 IIB–IVA stage cervical cancer patients was included. The recurrence rate in our study is slightly higher than CCRT joint surgery (16.7%), however, there was a decrease compared to CCRT alone (31.7%). Maybe it was the small sample size that led to such different results.

This study showed that pathological cancer residue was closely related to recurrence, and multivariate analysis of variance showed that cancer residue was an independent risk factor for postoperative recurrence, which was consistent with previous research results [7, 8]. Further study found that residual carcinoma associated with preoperative lesions in diameter, suggesting that patients with tumor lesions of large diameter were more likely to relapse. The higher recurrence rate may be associated with the lack of oxygen in the center of tumors, which leading to a decrease in the sensitivity of tumor cells to radiation and weakens the efficacy of radiotherapy [9]. In this study, postoperative pathological types were significantly correlated with postoperative recurrence. The recurrence rate of patients with simple squamous cell carcinoma was lower than other pathological types. This may because squamous cell carcinoma has higher radiosensitivity than other pathological types [10]. In this study, multivariate analysis of variance showed that age was also an independent risk factor for postoperative recurrence, which may be related to difference in hormone and immune levels in different age groups. However, it is still controversial whether age is related to recurrence and prognosis. Other previous studies [11] have pointed out that the degree of tumor differentiation can affect the sensitivity of tumor cells to radiation. However, this study did not suggest that the degree of differentiation of cervical cancer is related to local recurrence. This may be related to the small number of included cases and the uneven proportion of the degree of differentiation and a large sample study is needed for further validation. In this study, the incidence of postoperative complications was 16.3%, mainly due to mild myelosuppression and gastrointestinal reactions. The 2 cases of postoperative incomplete intestinal obstruction and intestinal fistula recovered after conservative treatment. By contrast to the previous studies (12.8–19.8%), the complication rate was within an acceptable range in our study [12, 13]. In addition, in this study, the time between the CCRT and surgery was compared in different group. By comparing the operative time, bleeding volume and postoperative complications, the interval > 6 weeks group was better than the group with interval ≤ 6 weeks and the result was statistically different. By analyzing the reason, the tissue was still in a state of hyperemia and edema after chemoradiotherapy, so the risk of bleeding and infection after operation in short-interval increased. Therefore, appropriately extending the time between CCRT and surgery can reduce intraoperative bleeding and facilitate postoperative recovery.

This study retrospectively analyzed the cervical cancer patients with IIB–IIIB stage who accepted extra-fascial hysterectomy and bilateral adnexectomy hysterectomy surgery after CCRT. The postoperative recurrence rate decreased than pure CCRT treatment and there was no obvious increase in postoperative complications. The joint is expected to improve the local control rate of locally advanced cervical cancer patients and improve the long-term curative effect.

This retrospective analysis is affected by many factors, and the sample size of this study needs to be expanded or better prospective study to explore the feasibility of this program.

## Conclusions

CCRT combined with surgery for stage IIB–IIIB cervical cancer was feasible, reduced the rate of postoperative recurrence and surgery complications were tolerated.

## Data Availability

The datasets used and/or analyzed during the current study are available from the corresponding authors upon reasonable request.

## Abbreviations

CCRT: Concurrent chemoradiation therapy
GTV: Gross tumor volume
CTV: Clinical target volume
PTV: Planning target volume
RTOG: Radiation Therapy Oncology Group
